# Particle-based simulation of charge transport in discrete-charge nano-scale systems: the electrostatic problem

**DOI:** 10.1186/1556-276X-7-135

**Published:** 2012-02-16

**Authors:** Claudio Berti, Dirk Gillespie, Robert S Eisenberg, Claudio Fiegna

**Affiliations:** 1 ARCES, University of Bologna and IUNET, Via Venezia 260, 47521, Cesena, Italy; 2 Department of Molecular Biophysics and Physiology, Rush University Medical Center, Chicago, IL, USA

## Abstract

The fast and accurate computation of the electric forces that drive the motion of charged particles at the nanometer scale represents a computational challenge. For this kind of system, where the discrete nature of the charges cannot be neglected, boundary element methods (BEM) represent a better approach than finite differences/finite elements methods. In this article, we compare two different BEM approaches to a canonical electrostatic problem in a three-dimensional space with inhomogeneous dielectrics, emphasizing their suitability for particle-based simulations: the iterative method proposed by Hoyles et al. and the Induced Charge Computation introduced by Boda et al.

## 1 Introduction

The investigation of the properties of a large variety of physical systems requires accurate computation of the electrostatic interactions among discrete fixed or mobile charges. This problem has been faced in a large number of cases including the analysis of electronic and optoelectronic devices [1-6], the investigation of fluid properties and the simulation of ion transport through membrane pores [7-12].

In nano-scale physical systems, some of the properties of the interacting bodies are strongly localized and can be approximated as Dirac delta functions. For example, the properties of spatially homogeneous ionic solutions have been investigated using the so called *primitive model *for the ions, considering them as hard spheres with finite radii and discrete point charges placed at the center of the spheres. On the other hand, in relatively large physical systems, the charge can be described by a continuous function of the spatial coordinates representing volume (or surface) charge density. The electrostatic problem requires the solution of Poisson's differential equation and boundary conditions. The numerical solution of Poisson's equation requires the discretization of the partial differential equation into a system of algebraic equations on a discretization grid of the simulation domain. In the case of nano-scale systems, the discrete nature of charges cannot be neglected. If the *primitive model *is adopted for the charges, the electrostatic interactions among them can be computed from Coulomb's law. If the system of interest includes different dielectric regions (or phases) characterized by different values of the permittivity and separated by abrupt boundaries, Coulomb's law is not enough. The charges on the boundary are not discrete. Indeed, every charge can interact significantly with every other charge through the boundary condition and a two-body treatment of electric forces (like Coulomb's law) is incomplete, and in that sense incorrect. In the boundary element method (BEM), the polarization effects associated with the discontinuity of permittivity at boundaries is accounted for by adding to the system the polarization charge induced over the boundary surface. This approach does not require the discretization of the whole simulation domain. It requires the discretization of boundary surfaces where discrete polarization charges are associated with discrete surface elements.

In this article, we compare the accuracy and computational speed of two different BEM approaches for the simulation of charged particles.

## 2 The electrostatic problem

We study a system of discrete point-charges embedded in an inhomogeneous medium composed of different dielectric regions separated by sharp boundaries . Poisson's equation is the fundamental law that, within the quasi-static approximation, links the charge density *ρ*(**r**) to the spatial distribution of the electric field **E**(**r**) and the electrostatic potential Φ(**r**). The discontinuity of permittivity occurring at the boundaries between different materials or phases is part of the system. Thus, we need to write Poisson's equation taking into account the relationship between the electric field **E**(**r**) and the polarization vector **P**(**r**), as:

(1) $$\varepsilon_{0}\nabla \cdot E\left(r\right)=\rho \left(r\right)-\nabla \cdot P\left(r\right)$$

where *ϵ*_0 _is the permittivity of free space. Since we deal with discrete charges, except on the boundary, *ρ*(*r*) = Σ_*k*_*q*_*k*_*δ*(**r **- **r**_*k*_) where *q*_k _and **r**_*k *_are the charge and the position of the *k*th point charge, respectively. The polarization charge density *h*(**r**) = -∇ · **P**(**r**) can be expressed as:

(2) $$h\left(r\right)=\frac{1-\varepsilon \left(r\right)}{\varepsilon \left(r\right)}\rho \left(r\right)-\varepsilon_{0}\frac{\nabla \varepsilon \left(r\right)}{\varepsilon \left(r\right)}\cdot E\left(r\right)$$

As described in [12], in the frame of the *primitive model*, polarization charges are present only at the dielectric boundaries, leading to an integral equation in *h*(**s**):

(3) $$h\left(s\right)+\frac{\Delta \varepsilon \left(s\right)}{4\pi \bar{\varepsilon }\left(s\right)}n\left(s\right)\cdot \int_{ℬ}\frac{s-s^{\prime }}{{\left|s-s^{\prime }\right|}^{3}}h\left(s^{\prime }\right)ds^{\prime }=-\frac{\Delta \varepsilon \left(s\right)}{4\pi \bar{\varepsilon }\left(s\right)}n\left(s\right)\cdot \underset{k}{\sum }\frac{q_{k}}{\varepsilon \left(r_{k}\right)}\frac{s-r_{k}}{{\left|s-r_{k}\right|}^{3}}$$

where Δ*ϵ*(**s**) and $\bar{\varepsilon }\left(s\right)$ are the change and the mean value of the dielectric constant in the normal direction **n**(**s**) evaluated at the boundary . When the integral equation 3 is solved for *h*(**s**) on the two-dimensional boundary , all source and induced charges of the system are known and the electrostatics can be evaluated everywhere in the space using superposition of Coulombic contributions from both source and induced charges. Therefore, the potential at a given position **r**_*U *_is:

(4) $$\text{Φ(}r_{U}\text{)=}\frac{1}{4\pi \varepsilon_{0}}\sum_{k}\frac{q_{k}}{\varepsilon (r_{k})\left|r_{U}-r_{k}\right|}+\frac{1}{4\pi \varepsilon_{0}}\int_{ℬ}\frac{h(s)}{\left|r_{U}-s\right|}ds$$

The evaluation of the induced charges in Equation 3 and of the potential in Equation 4 requires the evaluation of surface integrals over the dielectric boundaries. This task can be accomplished by *N*-point quadrature, dividing the dielectric boundary into a number *N *of surface elements that we call "tiles". Therefore, the solution of Poisson's equation is converted into the solution of the linear system of equations, ***A*h **= **b**, where ***A ***is a *N *× *N *matrix describing the mutual electrostatic interaction among the *N *surface elements, the vector **b **is the electric field impinging on each surface element and the vector **h **contains the polarization charges induced at the discretization tiles of the dielectric boundaries.

## 3 Boundary elements methods

In this study, two different BEM implementations are compared: the iterative method (ITER) introduced by Hoyles et al. [13] based on the study of Levitt [14] and the induced-charge computation (ICC) of Boda et al. [12].

ITER and ICC differ in the way they evaluate the induced charges on the dielectric boundary. ITER approach does not solve a linear system of equations, but evaluates the polarization charges using an algorithmic iterative procedure: at each step, the charge induced by each source/induced charge on the surface elements is evaluated; the procedure is recursively repeated in order to take into account the mutual electrostatic interaction among the polarization charges induced at the surface elements, resulting in a progressive refinement of the estimated-induced polarization charges until a convergence criterion is met. ICC solution is obtained by simultaneously solving the coupled equations derived by the discretization of Equation 3 over the discretized boundary. In both cases, the evaluation of the electrostatic potential at a given position (Equation 4) is performed via Coulomb's law and superposition with an integral form of Coulomb's law for the induced charge on the boundaries.

## 4 Results

**Figure 1:**
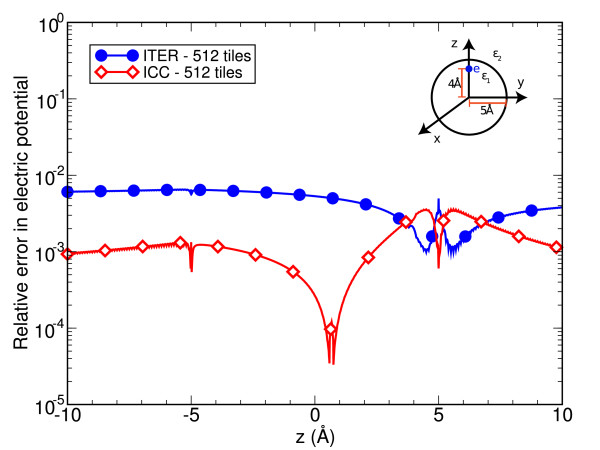
**(Color online) Sphere test case**. Relative error in the electric potential due to induced charges only along the diameter passing through the source charge. Results for both ITER (circles) and ICC (diamonds) are compared for the same number of boundary discretization tiles. The source charge is located at *z *= 4 Å.

The accuracy of ITER and ICC methods have been tested by applying them to the solution of a well-known electrostatic problem: a high-permittivity dielectric sphere (*ϵ*_1 _= 80) embedded in a low-permittivity dielectric medium (*ϵ*_2 _= 2) [12,15,16]. The sphere has 5 Å radius and an elementary point charge is located asymmetrically 4 Å off its center (inset of Figure 1). The dielectric boundary (i.e., the sphere surface) is divided into curved tiles, obtained by uniformly discretizing the spherical coordinates *θ *and *ϕ*. We solve the electrostatic problem by applying ICC and ITER to the same discretized domain, adopting an analytical description for the discretization tiles. In the ITER case, iterations are stopped when the relative update of the charge induced on each tile falls below *δ*_*i *_= 1 · 10^-4 ^[13].

Figure 1 shows the relative error, with respect to the analytic solution, for the electrostatic potential due to the surface-boundary induced polarization charges only, evaluated along the sphere diameter passing through the point charge. Both ITER and ICC yield very accurate results, limiting the relative error along the whole diameter to approximately 7 · 10^-3^.

**Figure 2:**
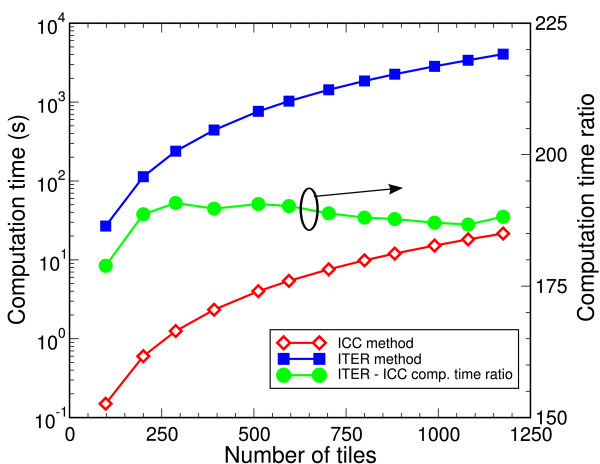
**(Color online) Sphere test case**. Left scale: computation time as a function of the number of tiles used to discretize the phase boundary for both ITER (squares) and ICC (diamonds). Right scale: ITER/ICC computation time ratio (circles). ICC works almost 200 times faster than ITER for any number of surface elements.

Figure 2 illustrates the computation time for the two methods as a function of the number of boundary discretization elements. ICC is considerably faster than ITER for any number of discretization elements. ICC solves the matrix equation ***A*h **= **b **with a matrix inversion and a matrix-vector multiplication. On the other hand, ITER adopts a complex algorithmic approach requiring, at each iteration, the (computationally expensive) evaluation of Coulombic contributions among the polarization charges induced at the discretization tiles [13,17]. In our implementations, the number of iterations needed to obtain the solution ranges from 80 to 140 depending on the number of tiles.

It is worth noting that the advantage of ICC is even more significant in numerical simulations in which the dielectric boundaries do not change during a simulation run. In such a case, the matrix must be inverted only once at the beginning of the simulation and then, at each time step, the electric forces can be evaluated on the basis of the charge distribution in the system through a *N *× *N*-matrix by *N-*vector multiplication. On the other hand, the large computation time needed by ITER makes the computation of electrostatics at run time difficult or even unfeasible.

To avoid long computation times, the electric forces in an ITER calculation are often stored in a number of look-up tables for different (possibly all) ion configurations in the system [18-20]. This approach has two major drawbacks: (i) the loss in accuracy due to the need to interpolate between look-up table entries; (ii) the practical impossibility to determine potential for asymmetric charge distributions due to extremely large memory requirements. ICC is not subject to these kind of problems since it can rapidly solve Poisson's equation for any charge distribution without lookup tables. Due to its accuracy and computation speed, ICC appears well suited for the simulation of nano-scale discrete-charge physical systems.

**Figure 3:**
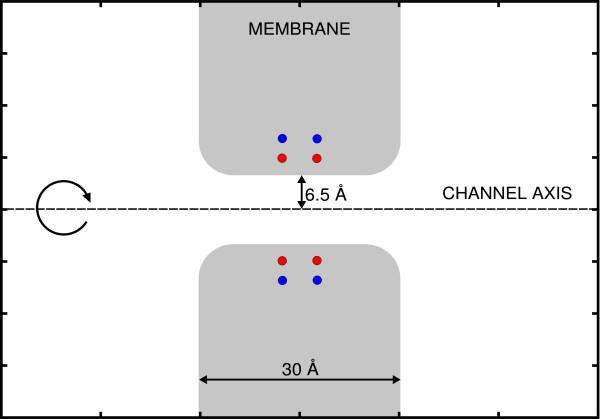
**(Color online) Toy model of ion channel**. Two ionic baths are connected through a cylindrical channel with round corners. The simulation domain is obtained rotating the 2D shape around the channel axis. Red and blue spheres represent the two rings of dipoles that mimic ion channel charged groups.

To provide more stringent and realistic tests, we checked ICC with a toy model of a cellular ion channel. The simulation domain is obtained rotating the 2D shape in Figure 3. Two water-like dielectric regions (*ϵ*_*W *_= 80) are connected via a cylindrical pore (6.5 Å radius) embedded in a membrane slab (*ϵ*_*M *_= 6) 30 Å wide. The dielectric boundary is discretized using 860 curved tiles defined analytically as described in the supplementary material of [12]. It is worth noting that ITER would provide results with the same accuracy of ICC, since it solves the same electrostatic problem, but computation time would be considerably larger. Furthermore, the accuracy of the solution depends only on the number of the discretization elements used to tile the boundary and is independent of the size and the width of the channel.

**Figure 4:**
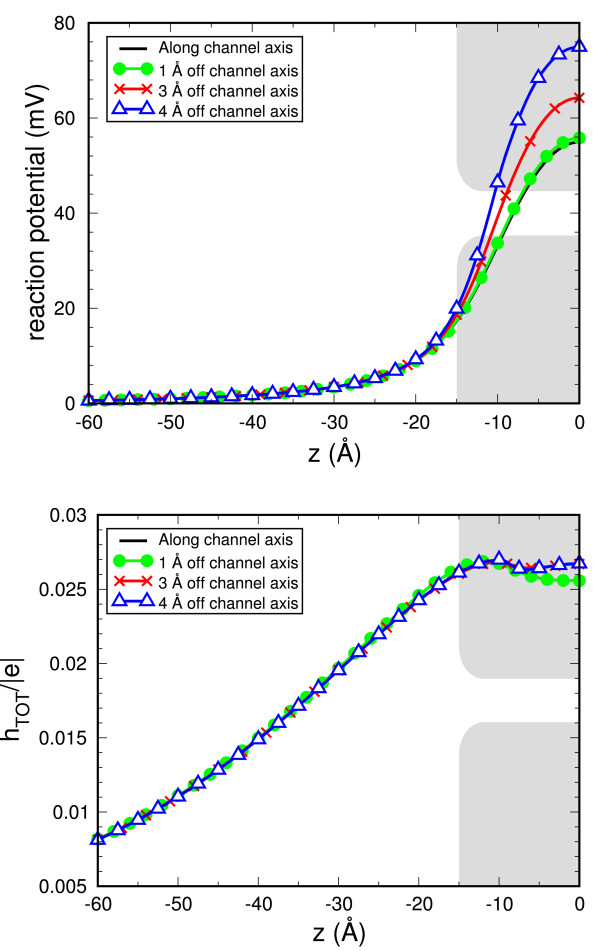
**(Color online) Toy model of ion channel**. **(a) **Reaction potential for a cation that moves along different trajectories parallel to the channel axis. A sketch of the channel profile is represented as a gray region. **(b) **Gauss's law check. The total charge induced on the dielectric boundary is plotted as a function of the position of the ion along the trajectories. The numerical error introduced by the computation is limited to 3% of an elementary charge.

We define "reaction potential" the electrostatic potential at a given point due to induced charges only:

(5) $$\Phi^{R}\left(r\right)=\frac{1}{4\pi \varepsilon_{0}}\overset{N}{\underset{i=1}{\sum }}\frac{h_{i}a_{i}}{\left|r-r_{i}\right|}$$

where *h*_*i*_, *a*_*i *_and **r**_i _are the induced charge density, the area and the position for the *i*th tile, respectively. Figure 4a graphs the reaction potential, 'felt' by a cation that moves along different trajectories parallel to the channel axis as it approaches the membrane center (*z *= 0). The symmetry of the simulation domain allows us to report results for only one half of the simulation domain. The reaction potential increases as the ion approaches the center of the membrane where it reaches a maximum. It increases as the ion moves a larger distance from the channel axis. An independent accuracy test consists in Gauss's law check: the total induced charge on the dielectric boundary must equal the total charge enclosed by the boundary. Since the only charge in this system is the ion that moves along the trajectories and it lies outside the dielectric boundary, the total charge enclosed by the boundary is 0. Therefore, the total charge induced on the boundary must be very close to 0. Figure 4b shows the total induced charge on the dielectric boundary as a function of the position of the ion along different trajectories. The total induced charge never exceeds a mere 3% of an elementary charge, but it is not zero. Since the simulation of realistic systems may involve up to thousands charges, in our view, the check of Gauss's law should be a feature of all calculations of electrostatics.

Finally, we tested ICC solutions in systems with asymmetrical charge distributions. We added two rings of 20 dipoles in the membrane to mimic ion channel charged groups. The dipoles are oriented such that their negative charges are placed 2 Å far from water-protein boundary, and the positive charges are placed 2 Å further inside the membrane. The orientation of the dipoles alter the potential profile in proximity of and inside the channel. No ions are present in the system.

**Figure 5:**
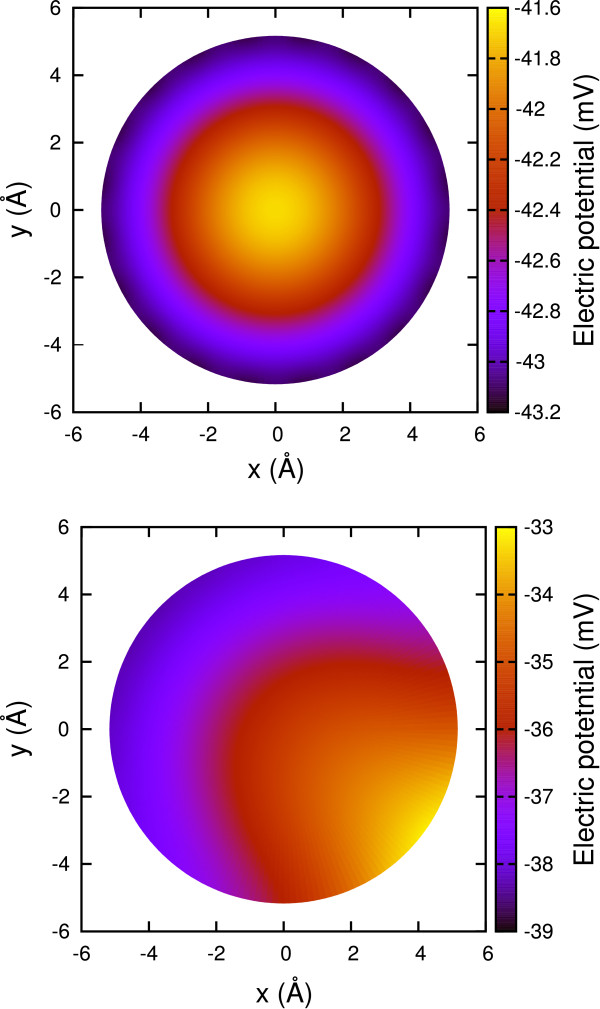
**(Color online) Toy model of ion channel**. **(a) **Potential map inside the cylindrical channel at *z *= 0 due to dipole rings embedded in the membrane. **(b) **Potential map at the same coordinate when 3 adjacent dipoles are switched off in each ring. The potential is the sum of both source charge and induced charge Coulombic contributions.

Figure 5a shows the potential map inside the cylindrical channel at the center of the membrane (*z *= 0). The potential at a given point is the sum of the Coulombic contribution from both discrete source and continuous induced charges. Due to the symmetry of the charge distribution, the potential features a radial symmetry reaching its maximum value at the channel axis (*x *= 0, *y *= 0). Then we broke the radial symmetry of the charge distribution by switching off three consecutive dipoles in each ring. The potential map reported in Figure 5b features a maximum displaced from channel axis and located close to the missing dipoles.

## 5 Conclusions

We compared ICC and ITER BEMs for the calculation of the electrostatics in discrete-charge systems. Our results show that both ICC and ITER feature high accuracy, but ICC is remarkably faster. This enables the evaluation of the electrostatics at run time during simulations. One can avoid the lookup tables to retrieve pre-calculated values that in our experience do not work well. ICC is therefore much more well suited for the simulation of nano-scale charged-particle systems such as ion channels or electronic devices.

## Competing interests

The authors declare that they have no competing interests.
